# Evolutional dynamics of 45S and 5S ribosomal DNA in ancient allohexaploid *Atropa belladonna*

**DOI:** 10.1186/s12870-017-0978-6

**Published:** 2017-01-23

**Authors:** Roman A. Volkov, Irina I. Panchuk, Nikolai V. Borisjuk, Marta Hosiawa-Baranska, Jolanta Maluszynska, Vera Hemleben

**Affiliations:** 1 Department of General Genetics, Center of Plant Molecular Biology (ZMBP), Eberhard Karls University of Tübingen, 72076 Tübingen, Germany; 2 Department of Molecular Genetics and Biotechnology, Yuriy Fedkovych University of Chernivtsi, Kotsiubynski str. 2, 58012 Chernivtsi, Ukraine; 3 Australian Centre for Plant Functional Genomics (ACPFG), The University of Adelaide, Hartley Grove, Urrbrae, SA 5064 Australia; 4 Current addres: School of Life Science, Huaiyin Normal University, 223300 Huaian, China; 5 Department of Plant Anatomy and Cytology, University of Silesia, 40032 Katowice, Poland

**Keywords:** Ribosomal DNA, Concerted evolution, Homogenization, Polyploidy, Solanaceae

## Abstract

**Background:**

Polyploid hybrids represent a rich natural resource to study molecular evolution of plant genes and genomes. Here, we applied a combination of karyological and molecular methods to investigate chromosomal structure, molecular organization and evolution of ribosomal DNA (rDNA) in nightshade, *Atropa belladonna* (fam. Solanaceae)*,* one of the oldest known allohexaploids among flowering plants. Because of their abundance and specific molecular organization (evolutionarily conserved coding regions linked to variable intergenic spacers, IGS), 45S and 5S rDNA are widely used in plant taxonomic and evolutionary studies.

**Results:**

Molecular cloning and nucleotide sequencing of *A. belladonna* 45S rDNA repeats revealed a general structure characteristic of other Solanaceae species, and a very high sequence similarity of two length variants, with the only difference in number of short IGS subrepeats. These results combined with the detection of three pairs of 45S rDNA loci on separate chromosomes, presumably inherited from both tetraploid and diploid ancestor species, example intensive sequence homogenization that led to substitution/elimination of rDNA repeats of one parent. Chromosome silver-staining revealed that only four out of six 45S rDNA sites are frequently transcriptionally active, demonstrating nucleolar dominance. For 5S rDNA, three size variants of repeats were detected, with the major class represented by repeats containing all functional IGS elements required for transcription, the intermediate size repeats containing partially deleted IGS sequences, and the short 5S repeats containing severe defects both in the IGS and coding sequences. While shorter variants demonstrate increased rate of based substitution, probably in their transition into pseudogenes, the functional 5S rDNA variants are nearly identical at the sequence level, pointing to their origin from a single parental species. Localization of the 5S rDNA genes on two chromosome pairs further supports uniparental inheritance from the tetraploid progenitor.

**Conclusions:**

The obtained molecular, cytogenetic and phylogenetic data demonstrate complex evolutionary dynamics of rDNA loci in allohexaploid species of *Atropa belladonna*. The high level of sequence unification revealed in 45S and 5S rDNA loci of this ancient hybrid species have been seemingly achieved by different molecular mechanisms.

**Electronic supplementary material:**

The online version of this article (doi:10.1186/s12870-017-0978-6) contains supplementary material, which is available to authorized users.

## Background

The realization that a large number of plant species, including many important industrial crops, evolved through circles of hybridization and/or polyploidization [1] has attracted intensive studies on the different aspects of polyploidy including mechanisms of polyploidy genome evolution [2]. Recent advances in plant genome and genomics research clearly demonstrate that hybridization/polyploidization involves intensive genomic rearrangements including exchanges between genomes, and loss or variation of gene copies and expression. These molecular processes are fundamental for species adaptive evolution and performance.

*Atropa belladonna* is a member of a small genus of ancient allopolyploid plants from the Solanaceae family with a history of medical applications due to its alkaloids, atropine and scopolamine [3, 4]. For a long time, its origin and taxonomic position remained an enigma. However, recent comparative DNA analysis has suggested that the genus *Atropa*, represented by 2 to 5 closely related species [5, 6] originated about 10 to 15 Myr (Million years) ago due to hybridization between a tetraploid species of Hyoscyameae and a now-extinct diploid species sister to the tetraploid lineage [6–8]. The uncertainty about one of the founding parents further complicates the tracing of species evolution. To learn more about the origin and genome evolution of this ancient natural polyploid, we studied genomic and molecular organization of the *Atropa belladonna* ribosomal DNA (rDNA).

Tandemly arranged repeated rDNA units found in genomes of all eukaryotes contain evolutionarily conserved sequences coding for ribosomal rRNAs and more rapidly evolving intergenic spacer regions (IGS). Because of its high copy representation in the genome and special arrangement of conserved coding regions linked with variable IGS, rDNA became an attractive focus for investigations of molecular evolution of repeated sequences and phylogenetic studies in different taxonomic groups [9–12]. Genomic loci representing 5S rDNA (5S rRNA gene plus the IGS) and 45S rDNA (genes coding for 18S, 5.8S, and 25S rRNAs and the spacer regions) are mostly arranged in head-to-tail tandem repeats. In contrast to the majority of repeated sequences, whose functions mostly remain unclear, activities of 5S and 45S rDNA genes are vital for organisms, providing rRNA species necessary for assembly of functional ribosomes, which account for more than 90% of total cellular RNA. In eukaryotes, the copy number (CN) of rDNA repeats is higher than is required for rRNA synthesis, and the redundant copies of rDNA are transcriptionally silenced [10, 13–15]. Transcriptionally active 45S rDNA loci (also known as nucleolus organizer regions, NORs) can be recognized by cytological chromosome analysis. Active loci produce nucleoli in interphase and secondary constriction (SC) regions of satellite-bearing chromosomes in metaphase [10, 13, 14]. Vascular plants often possess only single loci for both 5S and 45S rDNA, although multiple loci were also observed [10, 16–18].

Although numerous copies of rDNA repeats co-exist in the same genome, they tend to be nearly identical in many diploid species due to the process of sequence homogenization [19–21], i.e. individual copies of the repeated elements evolve not independently, but in a concerted manner [22, 23]. However, recently accumulated data suggested that a number of rDNA repeat units with different levels of sequence similarity can be simultaneously present in the same genome [24, 25]. This is especially true for species of hybrid origin (for review see [10, 26]), where the inheritance and evolution of rDNA can follow various scenarios. Often in the first generation hybrids, the 45S rDNA loci inherited from both parents remain structurally intact while enduring differential transcriptional silencing [13, 15, 27–30]. In ancient allopolyploid species, a more complicated picture is usually observed with uniparental inheritance and/or structural rearrangements of parental 45S rDNA. For example, in 0.2 Myr old natural allotetraploid *Nicotiana tabacum* all parental 45S rDNA loci were detected on chromosomes of ancestor diploids, *N. sylvestris* and *N. tomentosiformis*. However, the 45S rDNA repeats specific for *N. sylvestris* were almost completely eliminated and replaced by rearranged repeats of *N. tomentosiformis* [19, 31]. On the other hand, both parental 5S rDNA variants remained conserved in *N. tabacum* [32]. In contrast, in 4.5 Myr old *Nicotiana* alloploids of sect. *Repandae* both 5S and 45S rDNA loci and corresponding repeat variants of one parental species were not detected [33], indicating that the age of alloploid genome could be an important factor determining the character of rDNA in the hybrids.

Here, we present our data on the chromosomal localization/activity and molecular structure of 45S and 5S rDNA genes in *Atropa belladonna*. Based on uncovered specific loci representation and DNA sequences of 45S and 5S rDNA repeats, presumptive factors and mechanisms determining evolutionary dynamics of rDNA in polyploids are discussed.

## Methods

### Plant material

Seeds of *Atropa belladonna* (accession nos. 986 and 987) were obtained from the collection of the Botanical Garden, University of Tübingen.

### Chromosome analysis

Karyological analysis was performed as previously described [17]. Briefly, the primary root meristems of germinated seeds were pre-treated with 2 mM 8-hydroxyquinoline for 2 h at room temperature, fixed in ethanol-glacial acetic acid (3:1) and stored at −20 ° C. Excised roots were washed in 0.01 M citrate buffer (pH 4.8) prior to digestion in an enzyme mixture of 20% (v/v) pectinase (Sigma), 1% (w/v) cellulase (Calbiochem) and 1% (w/v) cellulase ‘Onozuka R-10’ (Serva) for 1.5–2 h at 37 ° C. Meristems were dissected out from root tips, squashed in drops of 45% acetic acid and frozen. After removal of coverslips, the preparations were post-fixed in 3:1 ethanol : glacial acetic acid, followed by dehydration in absolute ethanol and air-dried.

Double fluorescent staining with CMA (chromomycin A3) and DAPI (4′,6-diamidino-2-phenylindole) was performed according to [34]. Preparations were stained with CMA solution (0.5 mg/mL, Serva) for 1 h in the dark, briefly rinsed in distilled water and air-dried. Then slides were stained with DAPI solution (2 μg/mL, Serva) for 30 min in the dark, briefly rinsed in distilled water and mounted in antifade buffer (Citifluor, Ted Pella Inc.). Transcriptional activity of 45S rRNA genes was determined using silver staining following the method of [35]. Slides were treated with a borate buffer (pH 9.2) and air-dried. Then a few drops of freshly prepared 50% silver nitrate were applied to each preparation. Slides were covered with a nylon mesh and incubated in a humid chamber at 42 °C for 20 min, washed in distilled water, and air-dried. The karyological analysis was conducted on at least 10 slides of both the *A. belladonna* accessions 986 and 987. In each slide 10 metaphase plates were analysed.

### Fluorescence in situ hybridization (FISH)

For FISH, the following ribosomal DNA sequences were used as probes: 5S rDNA (pTa794) [36] labelled using PCR with tetramethyl-rhodamine-5-dUTP (Roche), and a 2.3-kb *Cla*I subclone of the 25S rDNA coding region of *Arabidopsis thaliana* [37] labelled by nick translation using digoxigenin-11-dUTP (Roche). The latter probe was used to determine the chromosomal localization of 45S rDNA. The following *in situ* hybridization of the probes and immunodetection of digoxigenated probe using FITC-conjugated anti-digoxigenin antibodies (Roche) were performed as described [17]. The fluorescence images were acquired using either an Olympus Camedia C-4040Z digital camera attached to a Leica DMRB epifluorescence microscope or a Hamamatsu C5810 CCD camera attached to an Olympus Provis AX epifluorescence microscope.

### Cloning and sequence analysis of 45S rDNA intergenic spacer (IGS)

Genomic DNA was isolated from leaves of 3-month-old plants using DNeasy Plant kit (Qiagen, Valencia, CA).

Our early restriction mapping experiments revealed that the 45S rDNA of *A. belladonna* possesses *Eco*RI recognition sites in the 18S and 25S rRNA coding regions, whereas no *Eco*RI site is present in the IGS [38] (Additional file 1: Figure S1). Therefore, *Eco*RI can be used for cloning of the complete IGS. Accordingly, DNA of *A. belladonna* (acc. no. 986) was digested with *Eco*RI, ligated into pBluescript SK and transformed into *E. coli* strain XL-blue. The library was screened for 45S IGS using ^32^P labelled DNA probe specific for 3′ end of 25S rRNA as describer earlier [19], and three clones - Ab-IGS-1S, −2S, −1L - were identified. One of the clones (Ab-IGS-1S), containing the complete IGS of the shorter size class of two rDNA repeats [38] was selected for detailed restriction mapping, generation of subclones and sequencing. In order to evaluate molecular heterogeneity of the 45S rDNA, the transcribed part of the 45S IGS, i.e. the 5′ ETS (external transcribed spacer, extended from presumptive transcription initiation site (TIS) to 18S rRNA coding region) was amplified by PCR for both accessions of *A. belladonna*, cloned and sequenced (clones Ab-ETS-4, −5, −6, −7, −8, −9, −10, −11, −12, −14, −15, −16, −18, −19, −21) as described earlier [21].

### Molecular analysis of 5S rDNA

The 5S rDNA units of *A. belladonna* were amplified by PCR using genomic DNA isolated from leaves of 3-month-old plants by DNeasy Plant kit (Qiagen, Valencia, CA), *Pfu* DNA polymerase (Thermo Fisher Scientific, Inc.) and primers Pr5S-L (5′-CAAT**GCGGCCGC**GAGAGTAGTACTAGGATGCGTGAC-3′) + Pr5S-R (5′-CATT**GCGGCCGC**TTAACTTCGGAGTTCTGATGGGA-3′) complimentary to the 5S rRNA coding region [20] were used for amplification. For subsequent cloning, *Not*I recognition sites (**GCGGCCGC**, printed in bold above) were added at the 5′ ends of both primers.

The reaction was performed in 50 μl of reaction mixture containing the following components: 0.1 μg of the genomic DNA, 1.0 U of DNA polymerase, 1 × PCR buffer, 4 mM MgCl_2_, 0.4 mM of each dNTPs, and 1 μM of each primer. The amplification was carried out at “standard” or “soft” conditions applying the following programs: (1) initial DNA polymerase activation at 95 °C, 4 min; (2) DNA denaturation at 94 °C, 40 s; (3) primer annealing at 57 °C, 45 s (standard) or at 54 °C, 90 s (soft); (4) DNA synthesis at 72 °C, 50 s (standard) or 20 s (soft); (5) amplification completion at 72 °C for 8 min. The total number of amplification cycles was 35. Optimization of the PCR soft conditions, which favours amplification of shorter 5S rDNA repeats (see Results for details), was carried out in the preliminary experiments.

PCR amplification of 5S rDNA was performed in triplicates and pooled PCR products of each accession were used for cloning. The fragments of different length were cut out from the gel, purified with a Gel Band Purification Kit (Qiagen), digested with *Not*I (Fermentas, Lithuania), ligated into *Eco*52I site of pLitmus 38 and transformed into *E. coli* strain XL-blue. Plasmid DNA isolation, restriction mapping and other standard procedures were carried out according to [39]. Inserts of selected clones were sequenced using the Big Dye Terminator Cycle Sequencing Kit and ABI Prism 310 sequencer (PE Applied Biosystems, USA). Sequence alignment was performed by CLUSTAL W method [40].

## Results

### Chromosomal organization of 5S and 45S rDNA

**Fig. 1:**
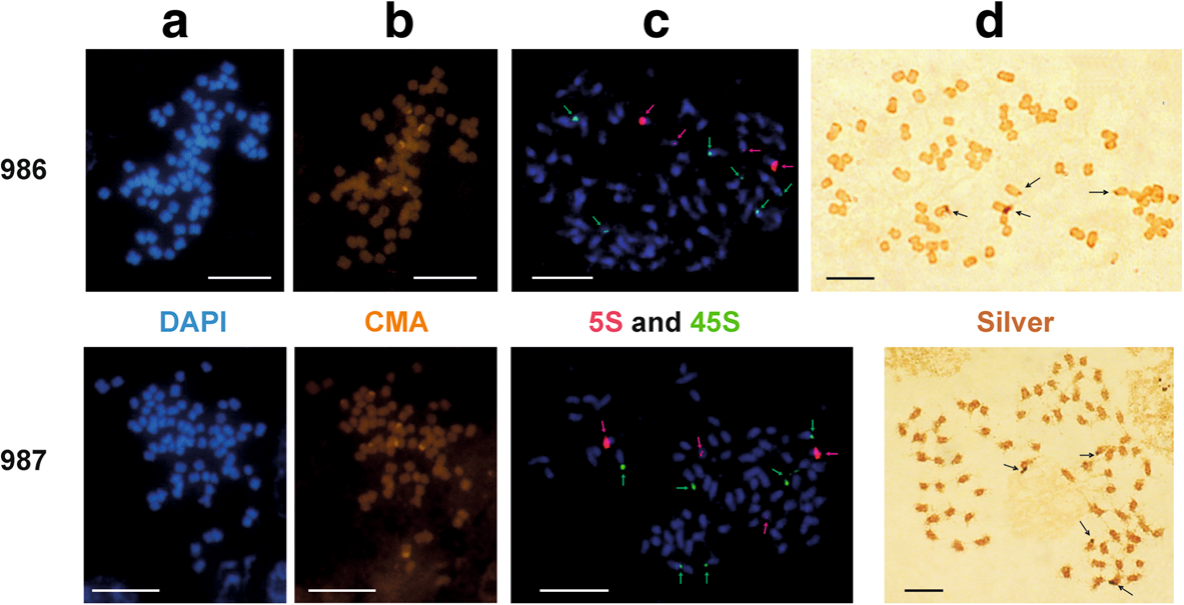
Karyological characterization of *Atropa belladonna* (accessions 986 and 987). **a** and **b** Double fluorescent staining with 4′,6-diamidino-2-phenylindole (DAPI) and chromomycin A3 (CMA), respectively; **c** Localization of 5S and 45S rDNA sequences on chromosomes; The chromosomes were stained by DAPI (*blue fluorescence*), hybridization signals of 5S (*red*) and 45S rDNA (*green*) are marked by *arrows*; **d** Active 45S rDNA (nucleolar organizing region, NOR) sites (*arrows*) in *Atropa belladonna* detected by silver staining; Scale bar, 10 μm

The chromosome number for *A. belladonna,* 72 chromosomes per somatic cell, was estimated by DAPI staining of root meristems. For 45S rDNA six distinct hybridization signals specific for 45S rDNA cluster were detected on separate chromosomes (Fig. 1)*.* Similarly, chromomycin A3 (CMA) staining produced six signals, two of which were relatively slight ones. Determination of 45S rDNA location was complemented by silver-staining, an indicator of transcriptional activity of these sites. Chromosome silver-staining resulted in four signals per cell, suggesting that only four 45S rDNA sites are transcriptionally active.

In contrast to 45S rDNA, only four 5S rDNA specific signals – two very strong and two weak – were detected at metaphase chromosomes of *A. belladonna* (Fig. 1)*.* After double FISH with rDNA probes, hybridization signals specific for 5S rDNA and 45S rDNA were observed on separate chromosomes, indicating that there is no co-localization of 5S and 45S rDNA gene clusters.

### Sequence organization of 45S rDNA intergenic spacer region in *A. belladonna*

**Fig. 2:**
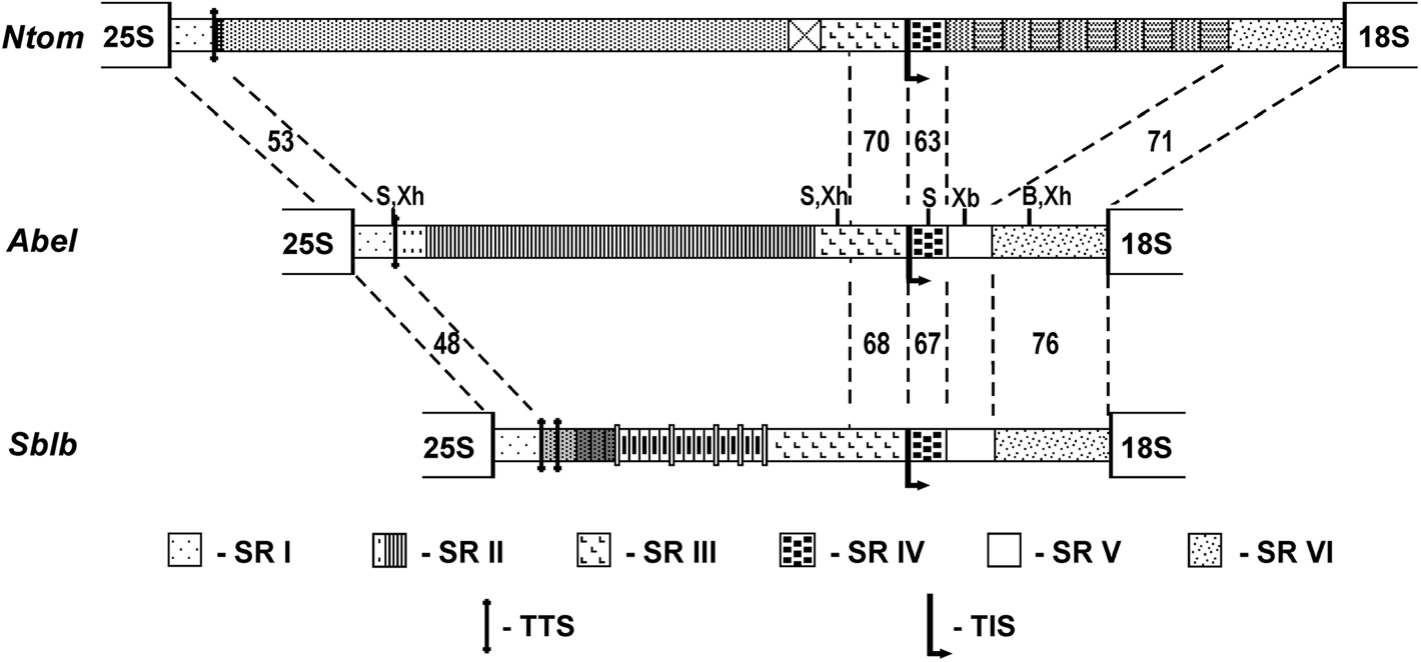
Organization and sequence similarity of the intergenic spacer (IGS) of 45S rDNA of *Atropa belladonna* (*Abel* - clone Ab-IGS-1S; Genbank Acc. No KF492694), *Solanum bulbocastanum* (*Sblb* – [27]) and *Nicotiana tomentosiformis* (*Ntom* – [19]). Percents of similarity for different structural regions (SR I – SR VI) of IGS are given. TIS: transcription initiation site; TTS: putative transcription termination site. Localization of restriction endonucleases recognition sites (B: *Bam* HI, EI: *Eco* RI, EV: *Eco* RV, S: *Sph* I, Xb: *Xba* I, Xh: *Xho* I) used for IGS mapping of *A. belladonna* rDNA is shown

**Fig. 3:**
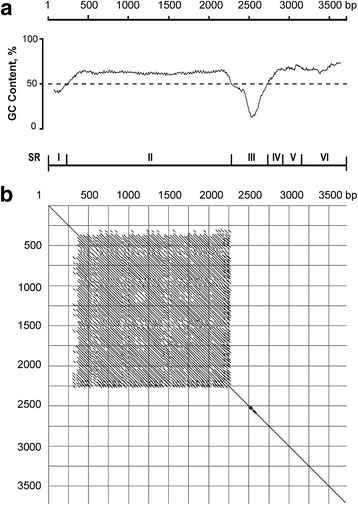
GC-content (**a**) and Harr-plot analysis (**b**) of nucleotide sequence of 45S IGS of *Atropa belladonna* (clone Ab-IGS-1S; Acc. No KF492694). A self-comparison of the IGS was made using the following parameters: window = 30, percentage = 70. Borders of structural regions (SR I – SR VI; see Fig. 2) are shown

In our cloning experiments we have isolated two short and one long DNA fragments containing IGS regions of the short and long variants of 45S rDNA repeats of *A. belladonna* (Additional file 1: Figure S1). Sequencing of one of the short clones (Ab-IGS-1S) showed the IGS region of 3710 bp. The sequence can be subdivided into six structural regions (SR I to SR VI; Fig. 2) according to Harr-plot analysis (Fig. 3), GC-content calculations and comparison with 45S rDNA IGS of other Solanaceae (see below).

The SR I (222 bp in length, 44.1% GC) consists of a unique sequence and exhibits moderate sequence similarity of 48 to 53% to representatives of distantly related Solanaceae species, *Solanum bulbocastanum* and *Nicotiana tomentosiformis* (Fig. 2). A pyrimidine-rich motif CCCTCCCCCTCC is present at the beginning of SR I (Additional file 2: Figure S2); similar motives were previously identified in the corresponding region of 45S rDNA in higher plants of different families [9, 41, 42]. At the 3′ end of SR I a GAGGTTTTT motif is located. From 1 to 4 copies of this sequence were found in representatives of distantly related genera of Solanaceae: *Nicotiana*, *Solanum* and *Capsicum* [19, 27, 31, 42, 43]. Obvious evolutionary conservation indicates functional importance of this motif, e.g. for transcription termination.

**Fig. 4:**
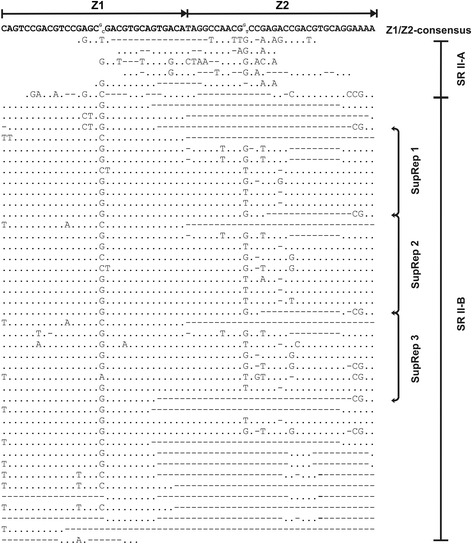
Molecular organization of structural region II (SR II) in the 45S IGS of *Atropa belladonna*. Alignment of individual Z-subrepeats and consensus sequences of Z1/Z2 subrepeats, borders of SR-IIA and -IIB and localization of Z-“super-repeats” (SupRep 1–3) are shown

The next IGS region, SR II (2055 bp in length, 61.8% GC) contains subrepeats (Figs. 2 and 3). This region can be subdivided in two sub-regions, SR II-A (162 bp) and -B (1893 bp). The SR II-B is composed of numerous copies of short subrepeats, two variants of which – Z1 (32 bp long) and Z2 (33 bp long) – can be distinguished (Fig. 4). In contrast, no perfect repeated elements, but only short fragments demonstrating similarity to Z-sub-repeats, were found in SR II-A.

All together, 37 perfect or partially deleted copies of Z1, 23 copies of Z2 and several short fragments of Z1/Z2-subrepeats were identified within SR II-B. The subrepeats are arranged as dimers Z1 + Z2 in the middle part of SR II-B, whereas the beginning and the end of SR II-B consist of Z1 subrepeats only. Further analysis revealed that some Z-subrepeats contain specific point mutations, which are periodically repeated within the SR II-B. Hence, in course of molecular evolution not only short motives and single Z-subrepeats, but also long arrays composed of several subrepeats were amplified. Such long blocks are shown as “super-repeats” in Fig. 4. A similar mode of amplification was described for C-subrepeats in SR II of *Nicotiana* [19]. Comparative restriction mapping of the cloned short and long IGS fragments demonstrated that two short clones appear to be identical whereas the long one differs by the length of the SR II by 0.75 kb (see Additional file 1: Figure S1 and Fig. 2). This difference is probably attributable to different numbers of Z-subrepeats.

The following SR III (464 bp long) is represented by a unique AT-rich (33% GC) sequence. It contains the putative promoter region including the transcription initiation site (TIS) at the 3′ end. A similar AT-rich region preceding the TIS has been found in *Solanum* [11, 27, 42], *Nicotiana* [19, 31], *Capsicum* [43] and other plant species [9, 41, 44].

The SR III can be further subdivided in two parts, A and B. The 185 bp-long SR III-A of *A. belladonna* exhibits a low similarity to 45S IGS of other Solanaceae, whereas the following 279 bp-long SR III-B and especially the region around the putative TIS are more conserved (Fig. 2 and Additional file 3: Figure S3). The two parts of SR III display a difference in GC-content that amounts to 44.9% for SR III-A vs. 25.1% for SR III-B. The difference is attributable to nine short GC-rich motives “imbedded” in the AT-rich sequence of SR III-A.

In the 45S IGS of *A. belladonna* no subrepeats are present down-stream of TIS. According to the comparison with the 45S IGS of other Solanaceae species, three regions – SR IV, V and VI – can be distinguished (Fig. 2).

The SR IV (185 bp long, 64.3% GC) of *A. belladonna* exhibits a moderate similarity with the corresponding IGS regions of *Solanum* and *Nicotiana* (Fig. 2 and Additional file 4: Figure S4). In the central part of this region a short, conserved element (CE: 41 bp) occurs, which demonstrates significant similarity – 76-80% – with other Solanaceae. Previously it was shown that CE is duplicated in the 45S IGS of potato *S. tuberosum* [21], and multiplicated in tomato *S. lycopersicum* and closely related species [11]. It was proposed that CE could be involved in transcription regulation, because differential transcription/silencing of parental 45S rDNA in interspecific hybrids of *Solanum* correlates with the number of CE [27]. In contrast to SR IV the following SR V (234 bp long, 68.0% GC) has no essential similarity with the 45S IGS of *Solanum* and *Nicotiana*; the level of sequence identity amounts to 58 and 41%, respectively.

Region SR VI adjacent to the 18S rRNA gene is 550 bp long (68.2% GC). The region exhibits comparatively high sequence similarity (71–76%) with the distantly related Solanaceae species (Fig. 2 and Additional file 5: Figure S5). Several segments of particularly high sequence identity were found in SR VI. These segments may be involved in regulation of transcription and/or processing of 45S rRNA.

In order to evaluate the level of intragenomic heterogeneity of individual repeats of 45S rDNA of *A. belladonna* we have amplified by PCR, cloned and sequenced the transcribed portion of 45S IGS (i.e., 5′ETS from presumptive TIS to 18S rRNA coding region). In total, 20 5′ETS clones were obtained and subjected to restriction mapping. For all clones, identical fragment patterns were obtained (data not shown). Afterwards, ten 5′ETS clones were randomly selected, sequenced and compared with the sequence of the complete 45S IGS described above. The results (Additional file 6: Figure S6) demonstrate that the level of sequence similarity between these eleven individual clones ranges from 98.2 to 100%. In the majority of clones, deviations from the consensus sequence were presented by 1 to 3 base substitutions, excepting clones Ab-ETS-9 and −10, which contain 11 and 10 substitutions, respectively. Also, 1- and 2-bp-long deletions and 1-bp-long insertion were found in the clones Ab-ETS-14, −16 and −12, respectively.

### Molecular organization of the 5S rDNA repeats

**Fig. 5:**
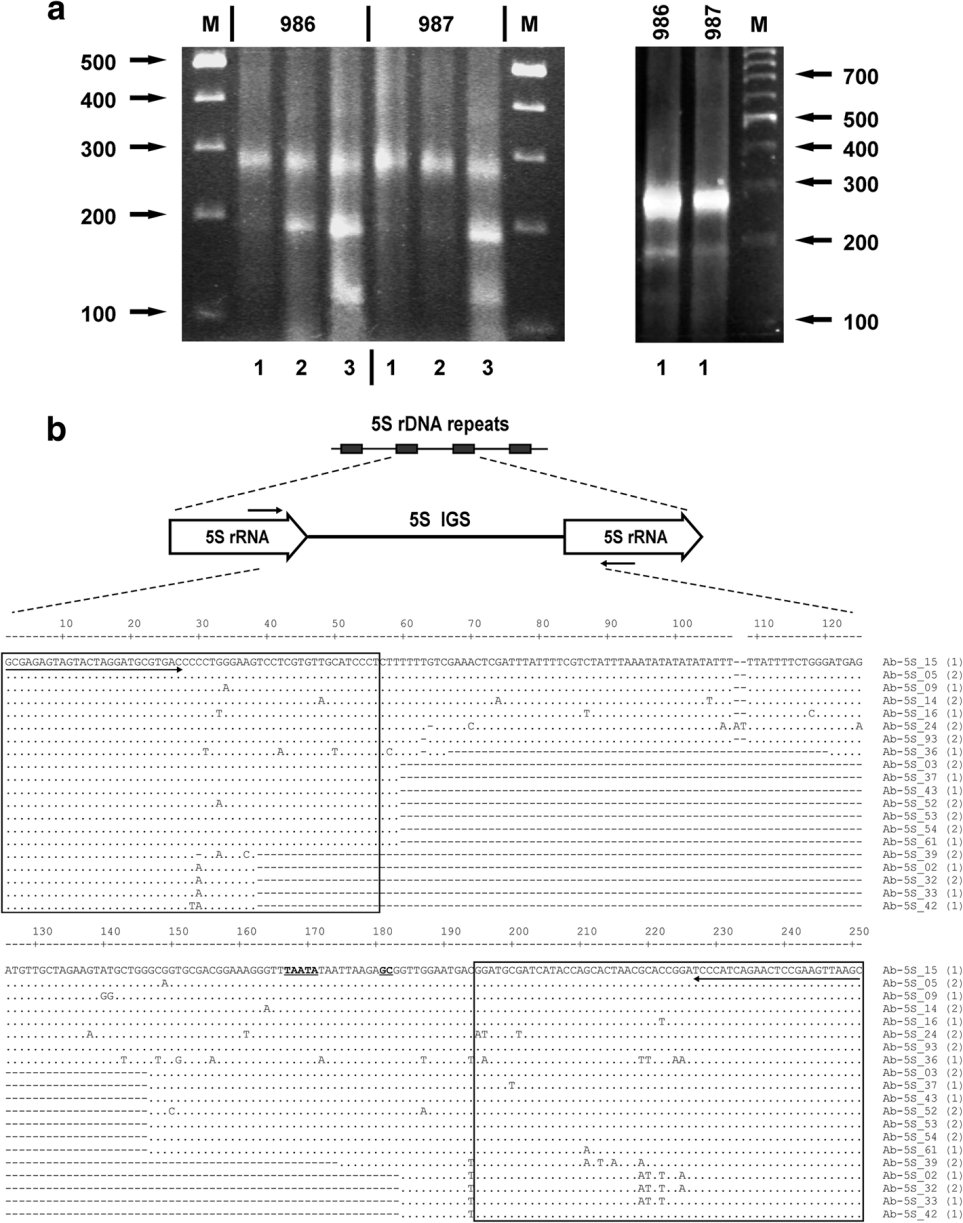
Molecular organization and polymorphisms of 5S rDNA repeats of *Atropa belladonna*. **a** Electrophoretic separation of 5S rDNA PCR products obtained for Acc. Nos 986 and 987; PCR amplification was performed (1) at standard conditions (see Methods), (2) at soft conditions, or (3) at soft conditions using increased (4 μM) concentration of primers; M, molecular weight marker; a ten-fold excess of the PCR product was loaded on gel in the right panel in comparison to the left panel. **b** Sequence comparison of 5S rDNA variants; Sequences of primers used for PCR amplification are marked by arrows, coding regions are shown as boxes and elements of presumptive external promoter are printed in bold underlined text; Numbers 1 and 2 shown in brackets are referred to Acc. Nos 986 and 987, respectively

Agarose gel separation of PCR products demonstrated that the main class of 5S rDNA repeats in *A. belladonna* has a length of about 260 bp (Fig. 5a). An additional shorter DNA fragment was detected when a large excess of sample was used for electrophoretic analysis (see Fig. 5a, right panel). The data show that the second minor class of 5S rDNA repeats, which has a length of about 180 bp, is present in the genome of *A. belladonna.* Evaluation of relative intensity of bands by the image analyzer showed that in accessions 986 and 987, respectively, from 5 to 7% and less than 2% of 5S rDNA repeats belong to the second minor class.

In order to clone different length variants of 5S rDNA, we performed optimization of PCR conditions (shortened elongation time, prolonged primer annealing under lower temperature, increased concentration of primers) to improve amplification of the shorter minor 180 bp-fragments. This resulted in preferential amplification of underrepresented 5S repeat variants. In particular, higher primer concentration led not only to improved generation of the 180 bp fragments, but also to amplification of a third class of 5S rDNA repeats, which have a length of about 120 bp (Fig. 5a, left panel, variant 3). This class remained undetectable under standard PCR amplification conditions. Hence, the 120 bp-repeats appear to be represented in the genome by very low CN.

Applying agarose gel electrophoretic fractionation, we have cloned PCR products representing all three size classes of 5S rDNA repeats. In total, for the two studied accessions of *A. belladonna*, 32 recombinant clones were isolated and, after determination of the insert lengths by restriction mapping, 20 of them were selected for sequencing. Comparison of the obtained sequences showed that the 5S repeats of *A. belladonna* can be classified into 3 groups: long (257–259 bp; clones pAb-5S-05, -09, −14, −15, −16, −24, −93), intermediate (171–203 bp; clones pAb-5S-03, −36, −37, -43, -52, -53, -54, -61), and short (113–121 bp; clones pAb-5S-02, −32, −33, −39, -42) repeats (Fig. 5b).

The long repeats are composed of the region coding for 5S rRNA and an IGS. Taking into account location of the primers used for PCR we calculated that as in other eukaryotes the length of the rRNA coding region is 120 bp. The length of 5S IGS of long repeats ranges from 137 to 139 bp, which is shorter compared to other representatives of Solanaceae, e.g. 165–229 bp in *Solanum* [20, 45] and 310–560 bp in *Nicotiana* [32, 46] species. In contrast to *Solanum,* no subrepeats were found in the central non-transcribed part of *A. belladonna* 5S IGS.

Sequence comparison showed that the long 5S rDNA repeats of both accessions of *A. belladonna* are very similar (96.4-99.6% of similarity, except one clone, pAb-5S_24 - see Fig. 5b). The minor differences are mainly due to occasional base substitutions and a few single nucleotide indels in the IGS. The 5S rRNA coding region of *A. belladonna* is identical to that of tomato *Solanum lycopersicum* and very similar to other Solanaceae (Additional file 7: Figure S7).

Similar to other plant species [20, 32, 47], an oligo-dT motif downstream of the coding region was found in the long 5S rDNA repeats of *A. belladonna.* These motives have been shown to function in Pol III transcription termination of the eukaryotic 5S rRNA genes [48]. Sequence comparison also revealed that in the long 5S IGS variant of *A. belladonna*, similar to other plant species [20, 32, 47], a TATA-like motif and a GC dinucleotide are located, respectively, at the positions −28 to −24 bp and −14 bp upstream of the coding region. These motives – together with the internal promoter elements – were proposed to form the Pol III transcription initiation site [48]. Thus, the long 5S rDNA repeats of *A. belladonna* contain the structurally normal 5S rRNA coding region and all known signals required for transcription initiation and termination. Therefore, they appear to be functionally active.

The intermediate 5S rDNA repeats contain a deletion (53–85 bp) in the central part of the IGS, compared to the long repeats. Nevertheless, they still possess all external promoter elements and the conserved 5S rDNA coding region (except the clone pAb-5S-36, which contains seven base substitutions in the sequenced fragments of the coding region). However, the intermediate repeats completely or partially (clone pAb-5S-36) lack the oligo-dT sequence required for transcription termination. These structural defects indicate that the intermediate repeats are non-functional, or that there is an alternative transcription termination option.

In the short 5S rDNA repeats, nearly the entire IGS is missing, as is 18 bp at the 3′ end of the coding region. Additionally, the short repeats accumulated several nucleotide substitutions in the rudimental fragment of 5S coding region, and a cytosine residue in position −1, which is required for transcription initiation [48], is changed into thymidine in all short clones sequenced. Accordingly, it looks probable that the short repeats represent pseudogenes.

We have calculated the number of base substitutions in individual 5S rDNA repeats compared to the consensus sequence and found that the three groups of repeats significantly differ by this parameter (Table 1 Specifically, the frequency of base substitutions is 1.43, 2.26 and 9.74 per 100 bp in long, intermediate and short repeats, respectively. Hence, the frequency of base substitutions appears to be about 1.6 and 6.8 times higher in intermediate and short repeats, respectively.

**Table 1:** Number of base substitutions in 5S rDNA of *Atropa belladonna*

Clone	Length	Transversions	Transitions		Total number of base substitutions
			C → T, G → A	T → C, A → G	
Group I: long repeats					
Ab-5S-05	199	0	1	0	1
Ab-5S-09	199	1	1	1	3
Ab-5S-14	199	2	2	0	4
Ab-5S-15	199	0	0	0	0
Ab-5S-16	199	2	2	0	4
Ab-5S-24	200	5	3	0	8
Ab-5S-93	198	0	0	0	0
Total for group I	1393	10 (50.0%)	9 (45.0%)	1 (5.0%)	20 (100%)
Group II: intermediate repeats					
Ab-5S-03	112	0	0	0	0
Ab-5S-36	144	6	9	1	16
Ab-5S-37	112	0	1	0	1
Ab-5S-43	112	0	0	0	0
Ab-5S-53	112	0	0	0	0
Ab-5S-52	112	1	2	0	3
Ab-5S-54	112	0	0	0	0
Ab-5S-61	112	0	1	0	1
Total for group II	928	7 (33.3%)	13 (61.9%)	1 (4.8%)	21 (100%)
Group III: short repeats					
Ab-5S-02	54	1	5	0	6
Ab-5S-32	53	1	5	0	6
Ab-5S-33	54	1	4	0	5
Ab-5S-39	62	3	4	0	7
Ab-5S-42	54	1	2	0	3
Total for group III	277	7 (25.9%)	20 (74.1%)	0	27 (100%)

*Note*: The number of base substitutions was calculated comparing sequences of individual clones and the consensus sequence. Lengths of clones are presented without primers used for PCR

Additionally, we have compared the frequency of different types of mutations and found that transitions amount to 50.0, 66.7 and 74.1% of all base substitutions in long, intermediate and short repeats, respectively (see Table 1). Remarkably, among 44 transitions detected, 42 were represented by C → T and G → A, which could be related to 5-methyl-cytosine deamination. Accordingly, it looks probable that the intermediate and short repeats were highly methylated for a long time, which resulted in preferential accumulation of respective transitions. Taken together, these data strongly support our proposition that the intermediate and short subrepeats represent pseudogenes. Thus, in ancient hexaploid *A. belladonna*, redundant 5S rDNA repeats did not evolve in a concerted manner. They appear to be gradually changed into pseudogenes and partially eliminated from the genome.

## Discussion

### Chromosome analysis and origin of *A. belladonna*

The small Old World polyploid genus *Atropa* possesses unique morphological traits and occupies an isolated taxonomic position within Solanaceae [5]. While the exact taxonomic position of *Atropa* is still debated, the majority of available data [8, 49] place this group within the tribe Hyascyameae, in spite of a marked difference in fruit morphology (fleshy berry-like fruits of *Atropa* versus dry capsules of other Hyascyameae). Within the tribe, *Atropa* is a sister group to the other six genera of Hyascyameae (*Anisodus, Atropanthe, Hyoscyamus, Physochlaina, Przewalskia*, and *Scopolia*). It is generally believed that *Atropa* species originated about 10 to 15 Myr ago through hybridization between a tetraploid species of Hyoscyameae and an extinct diploid progenitor related to the tetraploid lineage [6–8]. Therefore, genomic constitution of *Atropa* could be presented as EEH1H1H2H2, where E and H represent the genomes of **E**xtinct diploid and **H**yascyameae tetraploid parents, respectively.

The majority of karyology studies showed that *Atropa* species possess a karyotype of 2n = 72, although 2n = 50, 60 and 74 were also reported (see references presented in the Index to plant chromosome numbers at TROPICOS database - http://www.tropicos.org/Project/IPCN). Our results of chromosome analysis in *A. belladonna* are clearly consistent with the counts 2n = 72 (see Fig. 1).

The available cytogenetic data for the tribe Hyascyameae were differently interpreted in the available publications. Yuan et al. [7] propose for the section a base chromosome number x = 12 that is concordant with the well-supported taxonomic position of Hyascyameae within Solanoideae, which together with Nicotianoideae belong to the strongly supported monophyletic “x = 12” clade [49]. Accordingly, *Anisodus*, *Atropanthe* and *Scopolia* (2n = 48), *Przewalskia* (2n = 44), and *Physochlaina* (2n = 42) are considered as tetraploids, whereas *Hyoscyamus* possesses various chromosome numbers and ploidy levels [7, 50, 51]. In contrast, Tu et al. [52] believe that the basic chromosome number in the section is 6 (x = 6). According to this view, *A. belladonna* would be considered a dodecaploid. Chromosome staining with CMA and FISH experiments conducted in our study demonstrated that three loci (three pairs of sites) of 45S rDNA are present on six different chromosomes. This observation further supports the hexaploid constitution of *Atropa* as proposed by Yuan et al. [7] and revealed that *Atropa* possesses one 45S rDNA locus per chromosomal set.

Generally, the presence of a single chromosome pair with satellites is common in the family Solanaceae, especially in “x = 12” clade, and for several representatives of the clade the existence of single 5S and 45S rDNA loci was demonstrated by FISH ([27, 53–55] and references therein). Accordingly, multiple rDNA loci appear to be rare in “x = 12” clade and were found only in a few terminal clades that demonstrate intensive chromosomal evolution [50, 53, 55]. Thus, the available data show that *Atropa* most probably originated from parental species possessing single 5S and 45S rDNA locus per chromosomal set. Accordingly, six sites each of 5S and 45S rDNA could be anticipated in the modern allohexaploid *A. belladonna*. Our data reveal that the six sites of 45S rDNA are really present in *A. belladonna,* but only four 5S rDNA sites were found, demonstrating that two 5S rDNA sites were lost since the polyploid formation.

At the chromosomal level, contrasting evolutionary dynamics of plant 5S and 45S rDNA is a well-documented phenomenon. As demonstrated for several genera, 45S rDNA loci are more variable then 5S rDNA between closely related species, varieties and even individuals in terms of differences in size, number and loci locations [56–58]. Generally, in many plant species – both diploids and polyploids – the number of 5S loci is lower compared to 45S loci [16, 18], which could be used as another argument supporting different patterns of their molecular evolution.

### Molecular organization and evolution of 45S rDNA in *A. belladonna*

Considering a high similarity of the plant rDNA sequences coding for the 18S-5.8S-25S ribosomal RNAs [10], we concentrated our efforts on the analysis of evolutionary variable IGS. Based on restriction analysis and sequence characterization, the cloned *A. belladonna* IGS sequences represent two rDNA length variants of 9.4 and 10.2 kb revealed earlier by rDNA mapping experiments based on Southern blotting [38]. Sequencing of the short clone Ab-IGS-1S resulted in a 3710 bp long IGS fragment. Combining this IGS sequence with the 18S and 25S rRNA coding sequences of tomato and potato available in Genbank (Acc. Nos X51576, X67238, X13557) and ITS1-5.8S-ITS2 region of *Atropa* described by Uhink and Kadereit [6] we calculated that the length of *Atropa* rDNA unit is about 9.45 kb, which is very close to 9.4 kb, estimated earlier by Southern analysis. The larger cloned IGS fragment with a length of 6.51 kb, as estimated by restriction analysis, differs by the size of a region that contains Z-subrepeats up-stream of TIS (Additional file 1: Figure S1 and Fig. 2) and obviously corresponds to the longer 10.2 kb rDNA repeat. Detailed sequence characterization of the *A. belladonna* IGS revealed a structural organization similar to rDNA spacers of other *Solanaceae* species (Fig. 2), with specific functional subdivision into (i) a region of rRNA transcription termination, (ii) long block of sub-repeats, (iii) AT-rich region up-stream of TIS and (iv) the 5′ ETS adjacent to the 18S rRNA coding sequence.

Of special interest regarding the genome evolution in *A. belladonna* is a high level of IGS sequence homogenization between the two types of 45S rDNA length repeats. The short and long rDNA variants are situated at different sites according to their clear segregation [38] in somatic hybrids between *A. belladonna* and tobacco with incomplete chromosomal sets of both parents [59]. Comparison of 15 individual genomic DNA fragments of PCR amplified ETS clones demonstrated 98.2 to 100% sequence similarity. This high level of similarity was observed even in the central part of 5′ ETS, i.e. in SRV (Fig. 2), which is known to be more variable compared to other 45S rDNA regions. These data are also in a good agreement with the results on high sequence homogeneity in the ITS region of *Atropa* 45S rDNA [6] and comparable to intragenomic similarity of 45S rDNA repeats in diploid (98.4 to 99.9%) and polyploid (93.5 to 99.6%) species from the related genus *Solanum.* For comparison, the interspecific sequence similarities vary from 81 to 88% for representatives of distantly related *Solanum* species within sect. *Petota* [11, 22, 28].

The revealed high sequence similarity of 45S rDNA individual copies in allohexaploid *A. belladonna* leads to the suggestion that they originated from a single parent despite the location on all three sets of chromosome pairs. Previous studies showed, that in some cases supposedly uniparental rDNA inheritance has actually resulted from the intergenomic homogenization of parental 45S rDNA repeats [25, 26]. Indeed, we demonstrated that 45S rDNA repeats of allotetraploid *Nicotiana tabacum* structurally differ from the rDNA variants of parental diploid species, *N. sylvestris* and *N. tomentosiformis* [19, 31]. However, the 45S rDNA loci have the same chromosomal location in *N. tabacum* and in the parental species [60, 61]. We have also detected that in *N. tabacum,* 45S rDNA repeats originating from the maternal diploid species *N. sylvestris* were nearly completely substituted by structurally rearranged rDNA of the paternal diploid *N. tomentosiformis* [19, 27]. Later partial or complete conversion of parental 45S rDNA was reported for other alloploids of *Nicotiana* [33, 62]. Based on these considerations, we suggest that in *A. belladonna*, similar to *N. tabacum* [19], the repeats of one parent were overwritten or substituted by rDNA of the second parent following the process of sequence conversion.

The molecular conversion of 45S rDNA sequences in alloploids appears to be time dependent. As a rule, in the first generation of symmetric hybrids rDNA arrays of both parental forms are present [26, 27, 30]. However, the appearance of novel variants of 45S rDNA in first generation somatic hybrid between *N. tabacum* and *A. belladonna* [38] and in synthetic tobacco [62] shows that rearrangement of 45S rDNA units may start immediately after hybridization/polyploidization.

In young natural allotetraploid hybrids *Tragopogon mirus* and *T. miscellus,* which have been formed repeatedly during the past 90 years, partial loss of 45S rDNA of one parental diploid was observed, although the chromosome complements of parental diploids are additive and no chromosomal rearrangements were found [63]. In *Arabidopsis suecica*, a natural allotetraploid derived from *A. thaliana* and *A. arenosa*, one pair of *A. thaliana* NORs is missing. Similarly, in artificially obtained *A. suecica*-like allotetraploids, pairs of *A. thaliana* NORs are gained *de novo*, lost, and/or transposed to *A. arenosa* chromosomes [64]. Therefore, it seems likely that the loss of one pair of *A. thaliana* NORs observed in natural *A. suecica* was attributed to rapid chromosomal rearrangements during the next few generations after polyploidization. Similarly, in synthetic *Triticum/Aegilops* allopolyploids certain parental NOR loci were completely eliminated within several generations [30].

However, selective elimination of parental NORs and/or 45S rDNA variants may require much more time. In 0.2 Myr old *N. tabacum*, residual parental *sylvestris*-like rDNA ranges from 2 to 10% of total nuclear rDNA in different cultivars and amounts to 25% in feral tobacco [62], but three pairs of 45S rDNA loci are retained on the *sylvestris-*donated chromosomes. In 4.5 Myr old *Nicotiana* alloploids of sect. *Repandae,* only maternal 45S rDNA was found, and this completed diploidization was attributed to locus loss [33]. To the contrary, more ancient *Atropa,* whose age seems to be at least 10 Myr [6, 8], still possess six homogenized 45S rDNA loci of both parents, suggesting that not only time, but also some other factors could be responsible for the elimination or homogenization of redundant 45S rDNA loci.

IGS subrepeats have been proposed as one of the factors influencing homogenization of rDNA in plants. Comparing 45S rDNA molecular organization in *Brassica* and *Nicotiana*, Kovarik et al. [62] suggested that the presence of IGS subrepeats up-stream and down-stream of TIS in the *Nicotiana* species may be responsible for enhanced recombination potential of the rDNA, resulting in effective homogenization of the parental 45S rDNA in allopolyploids. In the IGS of *Atropa*, subrepeats were found only up-stream, but not down-stream of TIS (Fig. 2), nevertheless, the parental 45S rDNA is effectively homogenized. Thus, this process probably does not require the presence of two subrepeat regions in the IGS.

### Differential functional activity of the 45S rDNA loci in *A. belladonna*

In our karyological experiments we have found that mostly only two out of three 45S rDNA loci seem to be functionally active in *A. belladonna*. Accordingly, the question arises, which molecular mechanisms could be responsible for the differential activity of the 45S rDNA sites?

It is well known that in interspecific hybrids/allopolyploids 45S rDNA loci of one parent species are usually functionally active. This issue was originally described as nucleolar dominance (ND) by Navashin [65], and it was demonstrated later that at the molecular level ND means transcriptional silencing of 45S rDNA due to siRNA mediated differential methylation of cytosine, post-transcriptional modification of histone proteins and chromatin remodelling [13, 15, 27, 28, 30, 66]. It was also argued that differential activity of 45S rDNA in hybrids can be controlled by structural peculiarities of parental rDNA repeats (especially by that of IGS). Especially, it was found that in the IGS of *Xenopus* and *Arabidopsis,* the upstream subrepeats represent transcriptional enhancers [67, 68]. Later, it was also shown that in *Vigna* [69] and *Solanum* [27] the 45S rDNA repeats bearing more subrepeats down-stream of TIS appear to be more transcriptionally active. If no down-stream subrepeats are present in 45S rDNA, the variants possessing more subrepeats up-stream of TIS seem to be dominant (for review see [10, 13, 15, 26]). Considering these data, it is possible to suppose that four highly active sites of *Atropa* contain the long variant of 45S rDNA repeats, whereas the other two loci harbour the short one. Also, the differential transcription of 45S rDNA may be due to inactivation of complete NOR caused by local chromosomal context [70, 71], which may differ for 45S rDNA sites on *Atropa* chromosomes inherited from distantly related ancestors, i.e. for two E- versus four H-subgenomes.

For allotetraploids of *Nicotiana,* [29] it was found that the 45S rDNA loci participating in intergenomic conversion should be transcriptionally active, whereas transcriptionally silenced, highly methylated rDNA units escape homogenization, resulting in long-term persistence of both parental rDNA variants in the allopolyploid genome. Accordingly, it is possible to speculate that immediately after formation of the allohexaploid genome of *Atropa* the 45S rDNA loci of both parents were active, allowing intergenomic conversion. As a result, the 45S rDNA units of one parent were removed and substituted by rDNA of the second parent and later two loci (probably containing the short 45S rDNA variant and/or located on E-chromosomes) became less or not active. Presently, the mechanisms of presumptive differential activity of 45S rDNA variants in modern *A. belladonna* remain not yet understood and will be the focus of future research.

### Molecular evolution of *A. belladonna* 5S rDNA

In our karyological experiments four 5S rDNA specific signals – two very strong and two weak – were detected on *A. belladonna* chromosomes. Further molecular studies revealed that the 5S rDNA is represented by three length classes. The 5S rDNA repeats of the long class contain all structural elements required for transcription and, therefore, appear to be functionally active. In contrast, repeats of the intermediate and especially of the short class show structural defects like loss of signals involved in transcription initiation and even partial deletions of coding region. Also, they exhibit increased rates of base substitutions, i.e. typical features of non-functional pseudogenes. Remarkably, the increased rate of mutations seems to be preferentially attributed to the long-term increase of cytosine methylation.

The short and intermediate 5S rDNA repeats clearly differ in the character of structural defects and in the rate of base substitutions and, therefore, can be considered as early and late stages of functional rDNA transformation into pseudogenes.

Regarding the allohexaploid origin of *Atropa*, it was tempting to propose that the three structural classes of 5S rDNA repeats may represent three parental genomes and have to be located separately on three pairs of chromosomes. In order to clarify the origin of three 5S rDNA classes in *A. belladonna,* we have compared their IGS sequences, because it is well known that the region displays a very high rate of molecular evolution and may differ even between closely related species, e.g., in such genera as *Solanum* [20] and *Nicotiana* [32]. We have found (see Fig. 5) that three 5S rDNA classes present in the *A. belladonna* genome show high similarity in IGS and probably originate from the same parent. Hence, the 5S rDNA of the second parent was lost.

In order to assess the relative CN of 5S rDNA repeats of different length we have quantified the intensity of respective PCR products after electrophoretic separation and found that the long repeats are the most frequent in the 5S rDNA of *A. belladonna*. Respectively, comparing our karyological and molecular data it is possible to propose that two very large 5S rDNA sites contain potentially functional long repeats, whereas two weak sites contain intermediate repeats, i.e. slightly damaged pseudogenes. This proposition agrees well with earlier data that in other Solanacea 5S rDNA repeats are highly similar within an array [20, 32], and in other plants the similarity within an array is higher than between arrays [25].

The short repeats (i.e. hardly damaged pseudogenes) most likely exist as orphans outside of 5S rDNA loci and remained undetectable in karyological experiments due to their low CN. Alternatively, they could be interspersed with other repeats within 5S rDNA loci, but in this case it would be difficult to explain the high molecular evolution rate of short repeats. Similar to *A. belladonna*, 5S rDNA-related pseudogenes represented by minor sequence classes were found earlier in several species of different taxonomic groups, e.g., in *Solanum* [20], *Vigna* [12], *Rosa* [47] and *Thinopyrum* [25].

The origin of 5S rDNA was studied in several alloploids/hybrids; either bi- or uniparental inheritance was found [12, 20, 24, 32, 33, 64]. In the case of uniparental inheritance, elimination of 5S rDNA repeats of one parent was attributed to the loss of respective chromosomal loci, i.e. in contrast to 45S rDNA, no replacement of one parental 5S rDNA was observed. For instance, unchanged parental variants of 5S rDNA are still present in the genome of *N. tabacum* [32]. For *Sanquisorba*, it was found that duplicated 5S rDNA loci tend to have been lost after polyploidization, whereas 45S rDNA sites tend to have been conserved [72]. Similarly, Clarkson et al. [33] demonstrated that in recently formed (0.2 Myr old) alloploids of *Nicotiana* the number of loci equal the sum of those of their parents whereas in ancient (4.5 Myr old) alloploids the loss of 5S rDNA loci of one parent occurred. Regarding these data it could be anticipated that in 10 Myr old *A. belladonna* the 5S rDNA loci of one parent may have been eliminated. Supporting this assumption, our studies revealed that two chromosomal sets – probably originated from E-diploid – bear no 5S rDNA loci. Hence, all 5S rDNA repeats currently present in the *Atropa* genome seem to be derived from the H-tetraploid parent.

As mentioned above, the genomic constitution of allohexaploid *Atropa* can be presented as EEH1H1H2H2. Regarding the very high sequence similarity of GBSSI gene copies inherited from the Hyascyameae parent [7], it looks probable that two copies of the H-genome (i.e., H1 and H2) were very closely related. Accordingly, it could be expected that 5S rDNA repeats located on H1 and H2 chromosomes have to be very similar if not identical, and our sequencing data confirm this. However, in spite of the high similarity the evolutionary fate of the two sets of 5S rDNA repeats derived from the H-tetraploid parent seems to be different: one set remained functionally active, whereas the second one was transformed into pseudogenes.

Thus, elimination of redundant 5S rDNA repeats in *Atropa* occurred in two rounds: the loss of repeats donated by E-diploid is already completed whereas the removal of one set of H-tetraploid donated repeats is still in progress.

### Distinct patterns of molecular evolution of 5S and 45S rDNA during *Atropa* diploidization

Taken together, our results demonstrate that in an ancient allohexaploid *A. belladonna* parental chromosomal loci of 45S rDNA were conserved*,* but at the sequence level the 45S rDNA endured interchromosomal conversion resulting in elimination of rDNA repeats of one parental species. In contrast, 5S rDNA sequences of one parent were either simply deleted from the genome, or firstly converted into pseudogenes and then lost. Hence, homogenization of 5S and 45S rDNA was achieved by different means: partial deletion of 5S rDNA repeats versus interchromosomal conversion of 45S rDNA. These rearrangements resulted in marked difference at chromosomal level: the number of 5S loci was reduced whereas that of 45S rDNA was preserved in evolution.

The partial loss of 5S and 45S rDNA repeats in *Atropa* represents an example of genomic diploidization, a well-known phenomenon occurring during the evolution of polyploid organisms. This phenomenon embraces numerous functional and structural changes at different levels like loss of redundant gene expression, rearrangements of repeated genomic sequences, loss of chromosomes, etc. [73–75].

Earlier, several modes of rDNA molecular evolution were proposed [22, 23, 76]. The first of them is a concerted evolution, where rDNA arrays evolve in a concerted manner, resulting in high similarity of individual repeats. The mechanisms of sequence homogenization are not fully understood. Yet, unequal crossing-over, gene conversion and purifying selection were proposed as molecular mechanisms of concerted evolution [76–78]. This mode of evolution can explain intragenomic variation of 45S rDNA ITS2 in 66% of plant species [24]. In *Atropa*, concerted evolution seems to have occurred for 45S rDNA, resulting in high similarity of repeated units separately located at the loci of three chromosomal pairs.

The second mode is birth-and-death evolution, whereby repeated units endure cycles of duplications, divergence and partial loss. Birth-and-death evolution is the best model to explain ITS2 variation in 27% of plant species [24]. In *Atropa*, such a scenario appears to be realised for the 5S rDNA.

## Conclusions

The combined molecular, cytogenetic and phylogenetic data obtained in this study demonstrate complex evolutionary dynamics or rDNA loci in allohexaploid species of *Atropa belladonna*. Our study revealed the high level of rDNA sequence unification in this ancient hybrid species and showed different molecular mechanisms underlying sequence homogenization at the 45S and 5S rDNA loci. The 45S rDNA endured interchromosomal conversion resulting in elimination of rDNA repeats of one parental species. In contrast, 5S rDNA sequences of one parent were either eliminated from the genome, or converted into pseudogenes and then lost. These molecular events led to reduction of the 5S rDNA loci number on *A. bellabonna* chromosomes while the 45S loci were preserved. The presented data further contribute to better understanding of molecular processes underlying formation and evolution of nuclear genomes in polyploid plant species.

## Additional files

Additional file 1: Figure S1. Localization of restriction endonucleases recognition sites (B: *Bam* HI, EI: *Eco* RI, EV: *Eco* RV, S: *Sph* I, Xb: *Xba* I, Xh: *Xho* I) in the coding region (a) and IGS (b) of *Atropa belladonna* 45S rDNA. (JPG 2102 kb)
Additional file 2: Figure S2. Nucleotide sequence comparison of 45S IGS structural region I (SR I) of *Atropa belladonna* (Abel)*, Solanum bulbocastanum* (Sblb - Komarova et al. 2004) and *Nicotiana tomentosiformis* (Ntom - Volkov et al. 1999). Pyrimidine-rich motives present at the beginning of SRI in different Solanaceae species are boxed. Presumptive transcription termination signal GAGGTTTTTT at the end of SRI is shown in bold. (PDF 69 kb)
Additional file 3: Figure S3. Nucleotide sequence comparison of 45S IGS structural region III-B (SR III-B) of *Atropa belladonna* (Abel) and corresponding regions of *Solanum bulbocastanum* (Sblb) and *Nicotiana tomentosiformis* (Ntom). (PDF 40 kb)
Additional file 4: Figure S4. Nucleotide sequence comparison of 45S IGS structural region IV (SR IV) of *Atropa belladonna* (Abel) and corresponding regions of *Solanum bulbocastanum* (Sblb) and *Nicotiana tomentosiformis* (Ntom). (PDF 39 kb)
Additional file 5: Figure S5. Nucleotide sequence comparison of 45S IGS structural region VI (SR VI) of *Atropa belladonna* (Abel) and corresponding regions of *Solanum bulbocastanum* (Sblb) and *Nicotiana tomentosiformis* (Ntom). (PDF 43 kb)
Additional file 6: Figure S6. Nucleotide sequence comparison of individual copies of 5′ETS region of *Atropa belladonna* 45S rDNA. Sequence of presumptive TIS is presented in bold underlined. (PDF 152 kb)
Additional file 7: Figure S7. Nucleotide sequence comparison of 5′ (a) and 3′ (b) portions of 5S rDNA coding region of *Atropa belladonna* (Abel: this study, consensus sequence – see Fig. 5)*, Solanum lycopersicum* (Slyc: Genbank Acc. No X55697), *S. tuberosum* (Stub: X82780), *S. pinnatisectum* (Spnt: X82779) and *Petunia hybrida* (Phyb-A: X07930, Phyb-B: X07929, Phyb-C: X07928). Sequences of primers Pr5S-R (A) and Pr5S-L (B) used for *A. belladonna* 5S rDNA amplification are marked as arrows. (JPG 1600 kb)
