# Supplementary figures and images for: Evolutional dynamics of 45S and 5S ribosomal DNA in ancient allohexaploid Atropa belladonna
Source: BMC Plant Biol. 2017 Jan 23;17:21. doi: 10.1186/s12870-017-0978-6 (PMC5260122; doi:10.1186/s12870-017-0978-6)

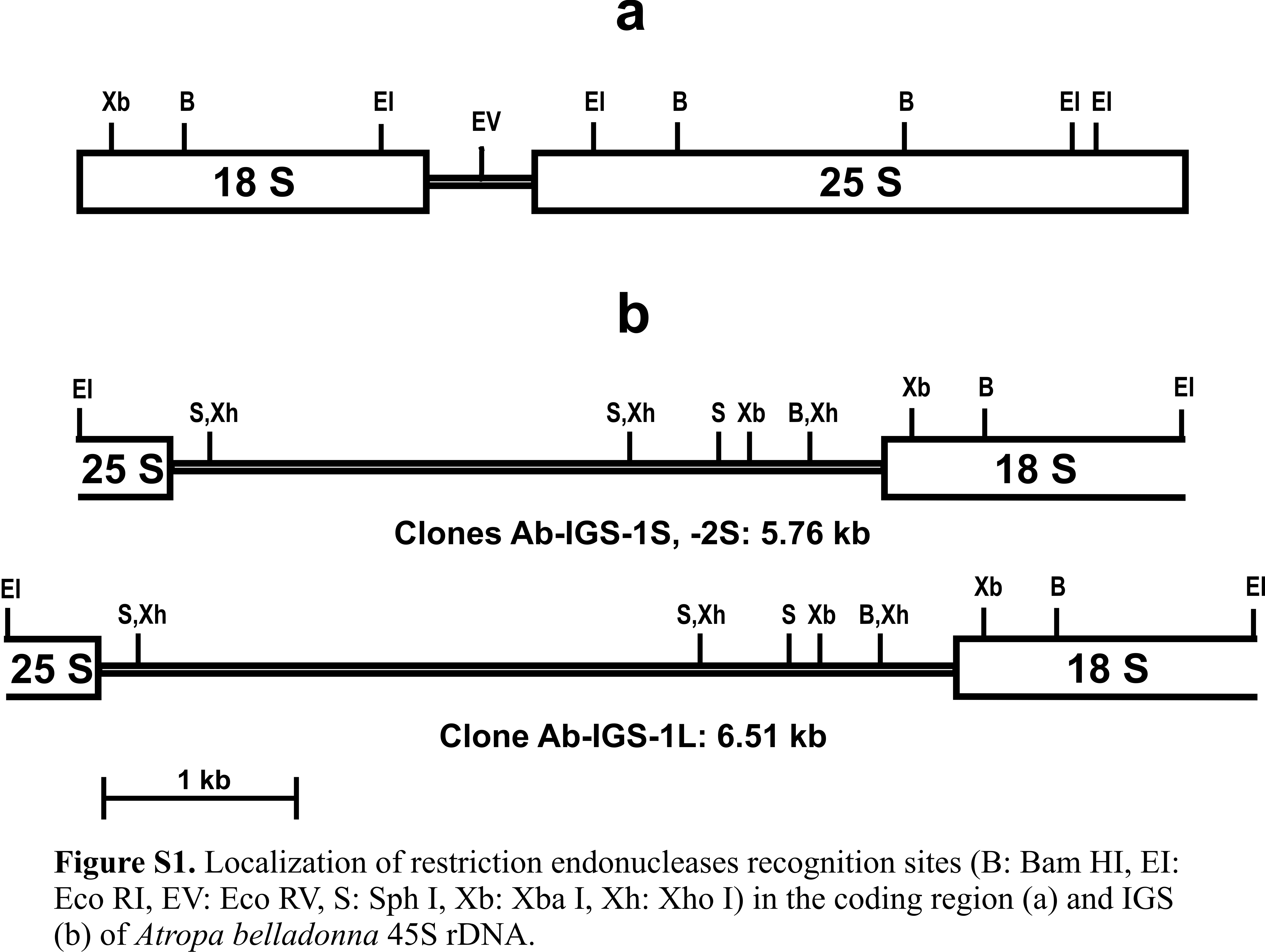

Supplement: Additional file 1: Figure S1. — Localization of restriction endonucleases recognition sites (B: Bam HI, EI: Eco RI, EV: Eco RV, S: Sph I, Xb: Xba I, Xh: Xho I) in the coding region (a) and IGS (b) of Atropa belladonna 45S rDNA. (JPG 2102 kb) [file 12870_2017_978_MOESM1_ESM.jpg]

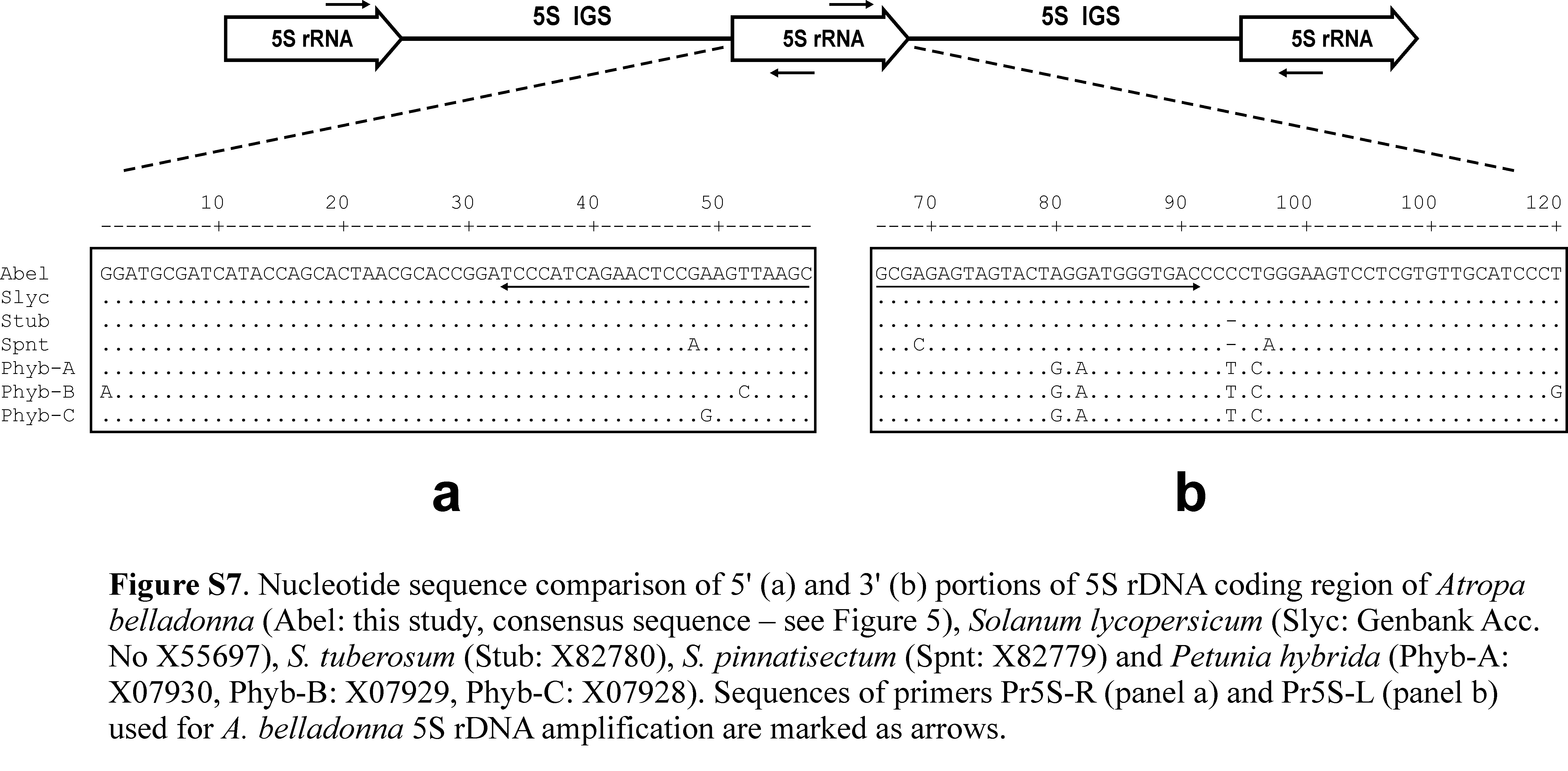

Supplement: Additional file 7: Figure S7. — Nucleotide sequence comparison of 5′ (a) and 3′ (b) portions of 5S rDNA coding region of Atropa belladonna (Abel: this study, consensus sequence – see Fig. 5), Solanum lycopersicum (Slyc: Genbank Acc. No X55697), S. tuberosum (Stub: X82780), S. pinnatisectum (Spnt: X82779) and Petunia hybrida (Phyb-A: X07930, Phyb-B: X07929, Phyb-C: X07928). Sequences of primers Pr5S-R (A) and Pr5S-L (B) used for A. belladonna 5S rDNA amplification are marked as arrows. (JPG 1600 kb) [file 12870_2017_978_MOESM7_ESM.jpg]
