# Supplementary material for: Evolutional dynamics of 45S and 5S ribosomal DNA in ancient allohexaploid Atropa belladonna
Source: BMC Plant Biol. 2017 Jan 23;17:21. doi: 10.1186/s12870-017-0978-6 (PMC5260122; doi:10.1186/s12870-017-0978-6)
Supplement: Additional file 2: Figure S2. — Nucleotide sequence comparison of 45S IGS structural region I (SR I) of Atropa belladonna (Abel), Solanum bulbocastanum (Sblb - Komarova et al. 2004) and Nicotiana tomentosiformis (Ntom - Volkov et al. 1999). Pyrimidine-rich motives present at the beginning of SRI in different Solanaceae species are boxed. Presumptive transcription termination signal GAGGTTTTTT at the end of SRI is shown in bold. (PDF 69 kb) [file 12870_2017_978_MOESM2_ESM.pdf]

```
             10        20        30        40        50        60
    ---------+---------+---------+---------+---------+---------+
485 -------------------CCCTCCCCCTCCGAACCCCTCAACTTCTATCTTCTTCGAAA  Abel
 75 CCCCCCCCACCTTCCCCCT...C.AA....AA.T..AA...-T.....A...T.CAA..G  Sblb
488 -------------------............ATT..AA....T..A..A..CT.......  Ntom

             70        80        90        100       110       120
    ---------+---------+---------+---------+---------+---------+
526 GGAGGTTCACAAG----TCGCTTACTTG---TGATATGCC--GCTAAGTGTTGAAAAGCT  Abel
134 .......T.TGTATTGC.T..A.C....GAAG..A.AAAAATA.C............TTA  Sblb
529 A......T.T--------TC...C....ATGCT....TATAT..C........G...ATC  Ntom

             130       140       150       160       170       180
    ---------+---------+---------+---------+---------+---------+
577 AAGTGTTCGAGAGACTAAGGCTAAGTGTATTTTATTCTATGATAAGACTAAGGCCGTCAC  Abel
194 .GTC.CG----.T.A...CATGCTAC..GAA--...TAC.AG.TT.C.GCTAAGT..TGA  Sblb
581 G.ACAAGTCC-TC.A..CCATGG.AC.....C....TGTGT..T.AGTGTTAAAT..G..  Ntom

             190       200       210       220       230       240
    ---------+---------+---------+---------+---------+---------+
637 TTGTGTAGGCAAACACATTGGCTAGATGAGTAGAC---GCATGCCATAATAATGTGGCCA  Abel
248 AC-.A..T.TT-CT.TGCC.ATC.A.AAGTCGTT.---------A...GCG...CCC.A.  Sblb
640 ...-TC.CTT.GT.GTT...TGGGA.A.GC.T.C.TAT.ACGCTA.ACCC...T.AA.TT  Ntom

             250
    ---------+---
694 CTCGAGGTTTTTT      Abel
297 TG.........        Sblb
699 A.A.........       Ntom
```

Figure S2 Nucleotide sequence comparison of 45S IGS structural region I (SR I) of *Atropa belladonna* (Abel), *Solanum bulbocastanum* (Sblb - Komarova et al. 2004) and *Nicotiana tomentosiformis* (Ntom - Volkov et al. 1999). Pyrimidine-rich motives present at the beginning of SRI in different Solanaceae species are boxed. Presumptive transcription termination signal **GAGGTTTTTT** at the end of SRI is shown in bold.
