# Supplementary material for: Evolutional dynamics of 45S and 5S ribosomal DNA in ancient allohexaploid Atropa belladonna
Source: BMC Plant Biol. 2017 Jan 23;17:21. doi: 10.1186/s12870-017-0978-6 (PMC5260122; doi:10.1186/s12870-017-0978-6)
Supplement: Additional file 3: Figure S3. — Nucleotide sequence comparison of 45S IGS structural region III-B (SR III-B) of Atropa belladonna (Abel) and corresponding regions of Solanum bulbocastanum (Sblb) and Nicotiana tomentosiformis (Ntom). (PDF 40 kb) [file 12870_2017_978_MOESM3_ESM.pdf]

```
                 10        20        30        40        50        60
     ---------+---------+---------+---------+---------+---------+
2947 ATTAATGATTTTCTTATGTTGGGTATTCTTCCTAACATTCTTATATTTTTTGAACATTTT  Abel
1822 ...TG...A...T.GG....A----A.T..ATA.TTT..-.G........CCT.TT.G..  Sblb
3908 ..-TGGA.A...TC.CCA...----..T..T...TAT..A.A...A....CTT.TT....  Ntom

                 70        80        90       100       110       120
     ---------+---------+---------+---------+---------+---------+
3007 CTAAATTT-TTAATTATTTTTT--AAATTT--AATTAATTATTATTAAAAAAATAATTTT  Abel
1877 A.T.....AC..C.A......CGG..T...CC..AA..AATAA.AATT.....G....G.  Sblb
3963 TGT.T...A...TG........--CGT...AT...A...AT............AT...A.  Ntom

                130       140       150       160       170       180
     ---------+---------+---------+---------+---------+---------+
3062 AT--AATAAAAAATATTATTTTCAATGCTTACAAGTCATTAACGAACGCATTATATCGTT  Abel
1937 T------G....C....T....AT..ATA..A........TGT.G.A..TAATGTGT...  Sblb
4021 T.TTTG.....T....C....A.....T...A........TGT...TAT....GC.AAG.  Ntom

                190       200       210       220       230       240
     ---------+---------+---------+---------+---------+---------+
3120 TGCACACGAAAACGTGCACGTTGGTGTTGTAATTCGCCATTATGATTCTCTGGCACAGGC  Abel
1991 ..T..CTT.G........TA..T.G......CA.GTT................A.A.AT.  Sblb
4081 ..T..TTCGGTTT.....TT...........CA.GT.T................CA.AA.  Ntom

                250       260       270       280
     ---------+---------+---------+---------+------
3180 ATGTCTACTCCTGCCACTTGGGTTTTTTTTTTTTAAGCATATATAA   Abel
2051 .............T...A...............-............   Sblb
4141 .............T...................A.GA.........   Ntom
```

Figure S3 Nucleotide sequence comparison of 45S IGS structural region III-B (SR III-B) of *Atropa belladonna* (Abel) and corresponding regions of *Solanum bulbocastanum* (Sblb) and *Nicotiana tomentosiformis* (Ntom).
