# Supplementary material for: Evolutional dynamics of 45S and 5S ribosomal DNA in ancient allohexaploid Atropa belladonna
Source: BMC Plant Biol. 2017 Jan 23;17:21. doi: 10.1186/s12870-017-0978-6 (PMC5260122; doi:10.1186/s12870-017-0978-6)
Supplement: Additional file 4: Figure S4. — Nucleotide sequence comparison of 45S IGS structural region IV (SR IV) of Atropa belladonna (Abel) and corresponding regions of Solanum bulbocastanum (Sblb) and Nicotiana tomentosiformis (Ntom). (PDF 39 kb) [file 12870_2017_978_MOESM4_ESM.pdf]

```
              10        20        30        40        50        60
     ---------+---------+---------+---------+---------+---------+
3226 GGGGGGTAGAGGTGTTGAA-GGCACTTCAGGGCGACTGTCGCC-GTTCGAGGCCAGCGCC  Abel
2096 ......C..........G.-....GACTGA......G.CA.G.-.GA..TCATGG...T.  Sblb
4187 ...................A.....C...A..T.CT..CT...T.GC......GG..ATG  Ntom

              70        80        90        100       110       120
     ---------+---------+---------+---------+---------+---------+
3284 CAGTGGGGCATGTGGGCGTGTAGTAGGGGCACACTCGTGGCATGCATGGCCTCGTCCAAG  Abel
2154 .T.....CTTA.........CT.CGT....G.-T.GA.............-.T....GT.  Sblb
4247 .-AC....G.C.CT...A..CG.CGT.....--T.G.G........C...-.T....GT.  Ntom

              130       140       150       160       170       180
     ---------+---------+---------+---------+---------+---------+
3344 CTACGCTAA-GGGTGTCAATCGAAAAACGCCGACGACGTCTCGCTGAGCGCCGGTAGTAG  Abel
2212 ......CGTT...C.A.T.CAA...C............G..T.TG.G...A.C..-..GC  Sblb
4303 .....TCCC-A.A...TCGC-A.CGCGT.T.AG.A.GTA....T-.T.T..TC......C  Ntom

              190
     ---------+
3403 GGCGGCGG     Abel
2271 C..T.A..GC   Sblb
4360 .T.T.GT.GT   Ntom
```

Figure S4 Nucleotide sequence comparison of 45S IGS structural region IV (SR IV) of *Atropa belladonna* (Abel) and corresponding regions of *Solanum bulbocastanum* (Sblb) and *Nicotiana tomentosiformis* (Ntom).
