# Supplementary material for: Evolutional dynamics of 45S and 5S ribosomal DNA in ancient allohexaploid Atropa belladonna
Source: BMC Plant Biol. 2017 Jan 23;17:21. doi: 10.1186/s12870-017-0978-6 (PMC5260122; doi:10.1186/s12870-017-0978-6)
Supplement: Additional file 5: Figure S5. — Nucleotide sequence comparison of 45S IGS structural region VI (SR VI) of Atropa belladonna (Abel) and corresponding regions of Solanum bulbocastanum (Sblb) and Nicotiana tomentosiformis (Ntom). (PDF 43 kb) [file 12870_2017_978_MOESM5_ESM.pdf]

```
              10         20         30         40         50         60         70
     ---------+---------+---------+---------+---------+---------+---------+
3161 CGTGTCT-AGCGGGACTTGCTCCGGACTCGCAACGTCGGCATCGGAAGCGGCGCTCGGCATATAATGCCG  Abel
2514 ..C.CAACG.....GA.A..AT...C..T....G.AA.......CGT...G..A......G.C......  Sblb
5799 ....C..-G.....G....TCTT..CT.T....T..T.....T..C.A.AA.A.AT...-..-CGC.T..  Ntom

              80         90        100        110        120        130        140
     ---------+---------+---------+---------+---------+---------+---------+
3230 GGCGTCGGGGCCGTTGTTTGGCACGCGACGACGAAGCATTTGGACGCACGGCGCGTGAGTGGTGTCGGGC  Abel
2584 ...........G.G..CCG...GT..AC.........TA..-C...T......A........A.T....  Sblb
5866 C.T..T.....T.....C-....T.TAT..G...G...AG..T...TG....A.A..........TC...  Ntom

             150        160        170        180        190        200        210
     ---------+---------+---------+---------+---------+---------+---------+
3300 GTGTGCGGTTAGGTTGGATCCCTGCT-CGAGCAGCGACCTCTTAGCCCGCACGCAGGGTCAGTCGGGGGA  Abel
2653 T..C......................T...........G..C..A......T..C.T...G..G.C..AT  Sblb
5935 T....T..C.................-T.T........G..C.........T..CATC......AT..C.  Ntom

             220        230        240        250        260        270        280
     ---------+---------+---------+---------+---------+---------+---------+
3369 CAAGCGCCGCAAGGGCTTGCCCGAAGTCGGTTTCCAGTGCTGCATACCTAATGCCCCGGCATTA-TCACG  Abel
2723 .......AATCTA......T.G..C......A...T............C.....G.G.......C..G..  Sblb
6004 .......AAATTA......TT..GC........T.T...T.................A.......-...A.  Ntom

             290        300        310        320        330        340        350
     ---------+---------+---------+---------+---------+---------+---------+
3438 TACGATCGGTCGCCTTTCGCCCGTCGCATCCAGCGTGCGGGCCGAACC-ACAAGCCGCTCCCGCGACCCA  Abel
2793 .G.A..................CC...GCT.GA..C.....G......C.G.........T....C....  Sblb
6073 C..A......T..........C....G.T.GA........GT.....-.A....T..AGTT.T.T....  Ntom

             360        370        380        390        400        410        420
     ---------+---------+---------+---------+---------+---------+---------+
3507 CTCCTTCCTCGCCTCGCCGCGCGATGGGGTGGTCCCGCGGAGGGCG-GTTCGGACTCTCGGATTCGGTAA  Abel
2863 .G.........TC..T..T....G..C.C.....-...AGC....-.C.G.A.T..............G  Sblb
6142 .G..A.......T...T..T......TCTA.....-AT.ACTA.TAT.C.....................  Ntom

             430        440        450        460        470        480        490
     ---------+---------+---------+---------+---------+---------+---------+
3576 ACGCAGCGGGCACGGGGCATTC------CCCCATCTGCCCTC--ACGA-CGCTCCCTACGAACGACGGTC  Abel
2931 ..................C...ACCGGCT..T..........GA....AT.....T.G..G.......C.  Sblb
6211 .....AT.....T....TC...ATTGGCT..T........---A.A..AT.....T......T.......  Ntom

             500        510        520        530        540        550        560
     ---------+---------+---------+---------+---------+---------+---------+
3637 GCGCCCGCGCTGCACCCGACCGCGCCCATCCGGGCG-GGTCTGGCTCACGCGGCGC-CGACGTCTGCGAG  Abel
3001 ....T...CT..GC....T........T.T......C..A.G..G...T.......G..G....GCT...  Sblb
6278 .T..T..TCT..G..TT.G...T.G..T.TG..T..--.C.AT.....T....T..-.......AAT...  Ntom

         566
     -----+
3705 GAACGC  3705   Abel
3071 ...T..  3076   Sblb
6345 ...T..  6350   Ntom
```

Figure S5 Nucleotide sequence comparison of 45S IGS structural region VI (SR VI) of *Atropa belladonna* (Abel) and corresponding regions of *Solanum bulbocastanum* (Sblb) and *Nicotiana tomentosiformis* (Ntom).
