# Supplementary material for: Evolutional dynamics of 45S and 5S ribosomal DNA in ancient allohexaploid Atropa belladonna
Source: BMC Plant Biol. 2017 Jan 23;17:21. doi: 10.1186/s12870-017-0978-6 (PMC5260122; doi:10.1186/s12870-017-0978-6)
Supplement: Additional file 6: Figure S6. — Nucleotide sequence comparison of individual copies of 5′ETS region of Atropa belladonna 45S rDNA. Sequence of presumptive TIS is presented in bold underlined. (PDF 152 kb) [file 12870_2017_978_MOESM6_ESM.pdf]

```
CTTGGGTTTTTTTTTTTTAAGCATATATAAGGGGGGTAGAGGTGTTGAAGGCACTTCAGG Majority
---------+---------+---------+---------+---------+---------+
         10        20        30        40        50        60
---------+---------+---------+---------+---------+---------+
............................................................ Ab-IGS-1S
............................................................ Ab-ETS-4
............................................................ Ab-ETS-5
..........................T................................. Ab-ETS-6
............................................................ Ab-ETS-7
............................................................ Ab-ETS-8
............................................................ Ab-ETS-9
............................................................ Ab-ETS-10
......................................A..................... Ab-ETS-11
................C........................................... Ab-ETS-12
.................-.......................................... Ab-ETS-14
...................G........................................ Ab-ETS-15
................--.......................................... Ab-ETS-16
............................................................ Ab-ETS-18
............................................................ Ab-ETS-19
............................................................ Ab-ETS-21

GCGACTGTCGCCGTTCGAGGCCAGCGCCC-AGTGGGGCATGTGGGCGTGTAGTAGGGGCA Majority
---------+---------+---------+---------+---------+---------+
         70        80        90        100       110       120
---------+---------+---------+---------+---------+---------+
.............................-.............................. Ab-IGS-1S
.............................-.............................. Ab-ETS-4
.............................-.............................. Ab-ETS-5
.............................-.............................. Ab-ETS-6
.............................-.............................. Ab-ETS-7
.............................-.............................. Ab-ETS-8
.............................-.............................. Ab-ETS-9
.............................-.............................. Ab-ETS-10
.......................C.....-.............................. Ab-ETS-11
.............................C.............................. Ab-ETS-12
.............................-.............................. Ab-ETS-14
.............................-.............................. Ab-ETS-15
.............................-.............................. Ab-ETS-16
.............................-.............................. Ab-ETS-18
.............................-.............................. Ab-ETS-19
.............................-.............................. Ab-ETS-21

CACTCGTGGCATGCATGGCCTCGTCCAAGCTACGCTAAGGGTGTCAACCGAAAAACGCCG Majority
---------+---------+---------+---------+---------+---------+
         130       140       150       160       170       180
---------+---------+---------+---------+---------+---------+
...............................................T............ Ab-IGS-1S
............................................................ Ab-ETS-4
............................................................ Ab-ETS-5
............................................................ Ab-ETS-6
............................................................ Ab-ETS-7
............................................................ Ab-ETS-8
...............................................T............ Ab-ETS-9
............................................................ Ab-ETS-10
............................................................ Ab-ETS-11
............................................................ Ab-ETS-12
............................................................ Ab-ETS-14
............................................................ Ab-ETS-15
............................................................ Ab-ETS-16
............................................................ Ab-ETS-18
............................................................ Ab-ETS-19
............................................................ Ab-ETS-21
```

```
ACGACGTCTCGCTGAGCGCCGGTAGTAGGGCGGCGGGCGGCATCGCTCGGGGCGGGCTGA Majority
---------+---------+---------+---------+---------+---------+
         190       200       210       220       230       240
---------+---------+---------+---------+---------+---------+
............................................................ Ab-IGS-1S
............................................................ Ab-ETS-4
............................................................ Ab-ETS-5
............................................................ Ab-ETS-6
............................................................ Ab-ETS-7
.................................................A.......... Ab-ETS-8
............................................................ Ab-ETS-9
.................................................A.......... Ab-ETS-10
............................................................ Ab-ETS-11
............................................................ Ab-ETS-12
............................................................ Ab-ETS-14
............................................................ Ab-ETS-15
............................................................ Ab-ETS-16
............................................................ Ab-ETS-18
............................................................ Ab-ETS-19
............................................................ Ab-ETS-21

CGGCTACGGACGGGACAAGGCGGTGCTTTCGTGTCGTGCGGTGAGTTCTAGACGCTTTTG Majority
---------+---------+---------+---------+---------+---------+
         250       260       270       280       290       300
---------+---------+---------+---------+---------+---------+
............................................................ Ab-IGS-1S
............................................................ Ab-ETS-4
............................................................ Ab-ETS-5
............................................................ Ab-ETS-6
............................................................ Ab-ETS-7
............................................................ Ab-ETS-8
............................................................ Ab-ETS-9
............................................................ Ab-ETS-10
............................................................ Ab-ETS-11
............................................................ Ab-ETS-12
............................................................ Ab-ETS-14
............................................................ Ab-ETS-15
............................................................ Ab-ETS-16
............................................................ Ab-ETS-18
............................................................ Ab-ETS-19
............................................................ Ab-ETS-21

TCTCTTGGCAAGCTCTAGGCGCCGGACGTACGGGGCTGAGCCGACGACCCGTCCGTCGCC Majority
---------+---------+---------+---------+---------+---------+
         310       320       330       340       350       360
---------+---------+---------+---------+---------+---------+
............................................................ Ab-IGS-1S
............................................................ Ab-ETS-4
............................................................ Ab-ETS-5
.....................................A...................... Ab-ETS-6
............................................................ Ab-ETS-7
.....................................A...................... Ab-ETS-8
............................................................ Ab-ETS-9
.....................................A...................... Ab-ETS-10
............................................................ Ab-ETS-11
............................................................ Ab-ETS-12
.....................................A...................... Ab-ETS-14
............................................................ Ab-ETS-15
............................................................ Ab-ETS-16
.....................................A...................... Ab-ETS-18
............................................................ Ab-ETS-19
.....................................A...................... Ab-ETS-21
```

```
GAACGTCCGCTGCGGGCGGTGTCGCCGTGCAGGCAGGCAGCTTGTCGTGGCTTCATCACC Majority
---------+---------+---------+---------+---------+---------+
         370       380       390       400       410       420
---------+---------+---------+---------+---------+---------+
............................................................ Ab-IGS-1S
............................................................ Ab-ETS-4
............................................................ Ab-ETS-5
............................................................ Ab-ETS-6
............................................................ Ab-ETS-7
............................................................ Ab-ETS-8
............................................................ Ab-ETS-9
...........T.....A.C........................................ Ab-ETS-10
............................................................ Ab-ETS-11
............................................................ Ab-ETS-12
............................................................ Ab-ETS-14
............................................................ Ab-ETS-15
............................................................ Ab-ETS-16
..............................................A............. Ab-ETS-18
............................................................ Ab-ETS-19
..........................A................................. Ab-ETS-21

TCTACCTCCCCGGGGGGCGGGGGGATTATCGTGTCTAGCGGGACTTGCTCCGGACTCGC Majority
---------+---------+---------+---------+---------+---------+
         430       440       450       460       470       480
---------+---------+---------+---------+---------+---------+
............................................................ Ab-IGS-1S
............................................................ Ab-ETS-4
............................................................ Ab-ETS-5
............................................................ Ab-ETS-6
............................................................ Ab-ETS-7
............................................................ Ab-ETS-8
............................................................ Ab-ETS-9
............A....G...........C.............................. Ab-ETS-10
............................................................ Ab-ETS-11
............................................................ Ab-ETS-12
............................................................ Ab-ETS-14
............................................................ Ab-ETS-15
............................................................ Ab-ETS-16
............................................................ Ab-ETS-18
............................................................ Ab-ETS-19
............................................................ Ab-ETS-21

AACGTCGGCATCGGAAGCGGCGCTCGGCATATAATGCCGGGCGTCGGGGCCGTTGTTTGG Majority
---------+---------+---------+---------+---------+---------+
         490       500       510       520       530       540
---------+---------+---------+---------+---------+---------+
............................................................ Ab-IGS-1S
............................................................ Ab-ETS-4
............................................................ Ab-ETS-5
............................................................ Ab-ETS-6
............................................................ Ab-ETS-7
............................................................ Ab-ETS-8
.........................................................C.. Ab-ETS-9
.........................................................C.. Ab-ETS-10
............................................................ Ab-ETS-11
............................................................ Ab-ETS-12
............................................................ Ab-ETS-14
............................................................ Ab-ETS-15
............................................................ Ab-ETS-16
............................................................ Ab-ETS-18
............................................................ Ab-ETS-19
............................................................ Ab-ETS-21
```

```
CACGCGACGACGAAGCATTTGGACGCACGGCGCGTGAGTGGTGTCGGGCGTGTGCGGTTA Majority
---------+---------+---------+---------+---------+---------+
         550       560       570       580       590       600
---------+---------+---------+---------+---------+---------+
............................................................ Ab-IGS-1S
............................................................ Ab-ETS-4
............................................................ Ab-ETS-5
............................................................ Ab-ETS-6
............................................................ Ab-ETS-7
............................................................ Ab-ETS-8
.G.......................................................... Ab-ETS-9
.G.......................................................... Ab-ETS-10
............................................................ Ab-ETS-11
............................................................ Ab-ETS-12
............................................................ Ab-ETS-14
.............G.............................................. Ab-ETS-15
............................................................ Ab-ETS-16
............................................................ Ab-ETS-18
............................................................ Ab-ETS-19
............................................................ Ab-ETS-21

GGTTGGATCCCTGCTCGAGCAGCGACCTCTTAGCCCGCACGCAGGGTCAGTCGGGGGACA Majority
---------+---------+---------+---------+---------+---------+
         610       620       630       640       650       660
---------+---------+---------+---------+---------+---------+
............................................................ Ab-IGS-1S
............................................................ Ab-ETS-4
............................................................ Ab-ETS-5
............................................................ Ab-ETS-6
............................................................ Ab-ETS-7
............................................................ Ab-ETS-8
............................................................ Ab-ETS-9
............................................................ Ab-ETS-10
............................................................ Ab-ETS-11
............................................................ Ab-ETS-12
............................................................ Ab-ETS-14
............................................................ Ab-ETS-15
............................................................ Ab-ETS-16
............................................................ Ab-ETS-18
...........................................C................ Ab-ETS-19
............................................................ Ab-ETS-21

AGCGCCGCAAGGGCTTGCCCGAAGTCGGTTTCCAGTGCTGCATACCTAATGCCCCGGCAT Majority
---------+---------+---------+---------+---------+---------+
         670       680       690       700       710       720
---------+---------+---------+---------+---------+---------+
............................................................ Ab-IGS-1S
............................................................ Ab-ETS-4
............................................................ Ab-ETS-5
............................................................ Ab-ETS-6
............................................................ Ab-ETS-7
............................................................ Ab-ETS-8
............................................................ Ab-ETS-9
............................................................ Ab-ETS-10
............................................................ Ab-ETS-11
............................................................ Ab-ETS-12
............................................................ Ab-ETS-14
............................................................ Ab-ETS-15
............................................................ Ab-ETS-16
............................................................ Ab-ETS-18
............................................................ Ab-ETS-19
............................................................ Ab-ETS-21
```

```
TATCACGTACGATCGGTCGCCTTTCGCCCGTCGCATCCAGCGTGCGGGCCGAACCACAAG Majority
---------+---------+---------+---------+---------+---------+
         730       740       750       760       770       780
---------+---------+---------+---------+---------+---------+
............................................................ Ab-IGS-1S
............................................................ Ab-ETS-4
............................................................ Ab-ETS-5
............................................................ Ab-ETS-6
............................................................ Ab-ETS-7
............................................................ Ab-ETS-8
......................................C..................... Ab-ETS-9
............................................................ Ab-ETS-10
............................................................ Ab-ETS-11
............................................................ Ab-ETS-12
............................................................ Ab-ETS-14
............................................................ Ab-ETS-15
............................................................ Ab-ETS-16
............................................................ Ab-ETS-18
............................................................ Ab-ETS-19
............................................................ Ab-ETS-21

CCGCTCCCGCGACCCACTCCTTCCTCGCCTCGCCGCGCGATGGGGTGGTCCCGCGGAGGG Majority
---------+---------+---------+---------+---------+---------+
         790       800       810       820       830       840
---------+---------+---------+---------+---------+---------+
............................................................ Ab-IGS-1S
............................................................ Ab-ETS-4
............................................................ Ab-ETS-5
............................................................ Ab-ETS-6
...............G............................................ Ab-ETS-7
............................................................ Ab-ETS-8
..................T.........T......T........................ Ab-ETS-9
............................................................ Ab-ETS-10
............................................................ Ab-ETS-11
............................................................ Ab-ETS-12
............................................................ Ab-ETS-14
............................................................ Ab-ETS-15
............................................................ Ab-ETS-16
............................................................ Ab-ETS-18
............................................................ Ab-ETS-19
............................................................ Ab-ETS-21

CGGTTCGGACTCTCGGATTCGGTAAACGCAGCGGGCACGGGGCATTCCCCCATCTGCCCT Majority
---------+---------+---------+---------+---------+---------+
         850       860       870       880       890       900
---------+---------+---------+---------+---------+---------+
............................................................ Ab-IGS-1S
............................................................ Ab-ETS-4
............................................................ Ab-ETS-5
............................................................ Ab-ETS-6
............................................................ Ab-ETS-7
............................................................ Ab-ETS-8
.A....................................................C....G Ab-ETS-9
............................................................ Ab-ETS-10
............................................................ Ab-ETS-11
..A......................................................... Ab-ETS-12
............................................................ Ab-ETS-14
............................................................ Ab-ETS-15
............................................................ Ab-ETS-16
............................................................ Ab-ETS-18
............................................................ Ab-ETS-19
............................................................ Ab-ETS-21
```

```
CACGACGCTCCCTACGAACGACGGTCGCGCCCGCGCTGCACCCGACCGCGCCCATCCGGG Majority
---------+---------+---------+---------+---------+---------+
         910       920       930       940       950       960
---------+---------+---------+---------+---------+---------+
............................................................ Ab-IGS-1S
............................................................ Ab-ETS-4
............................................................ Ab-ETS-5
............................................................ Ab-ETS-6
............................................................ Ab-ETS-7
............................................................ Ab-ETS-8
A........................................................... Ab-ETS-9
............................................................ Ab-ETS-10
............................................................ Ab-ETS-11
............................................................ Ab-ETS-12
............................................................ Ab-ETS-14
............................................................ Ab-ETS-15
............................................................ Ab-ETS-16
............................................................ Ab-ETS-18
............................................................ Ab-ETS-19
............................................................ Ab-ETS-21

CGGGTCTGGCTCACGCGGCGCCGACGTCTGCGAGGAACGC                     Majority
---------+---------+---------+---------+
         970       980       990       1000
---------+---------+---------+---------+
........................................                     Ab-IGS-1S
.........A..............................                     Ab-ETS-4
.........A..............................                     Ab-ETS-5
........................................                     Ab-ETS-6
.........A..............................                     Ab-ETS-7
........................................                     Ab-ETS-8
......C.................................                     Ab-ETS-9
........................................                     Ab-ETS-10
.........A..............................                     Ab-ETS-11
.........A..............................                     Ab-ETS-12
........................................                     Ab-ETS-14
.........A..............................                     Ab-ETS-15
.........A..............................                     Ab-ETS-16
........................................                     Ab-ETS-18
.........A..............................                     Ab-ETS-19
........................................                     Ab-ETS-21
```

Figure S6. Nucleotide sequence comparison of individual copies of 5'ETS region of *Atropa belladonna* 45S rDNA. Sequence of presumptive TIS is presented in bold underlined.
